# Color Stability of Ocular Prostheses Subjected to Short-Term Tropical Weathering: A Digital Photography Analysis

**DOI:** 10.7759/cureus.115866

**Published:** 2026-09-06

**Authors:** Nafij Bin Jamayet, James Dudley, Wan P Tee, Toh S Yin, Naveen Chabra, Spoorthi Ravi Banavar

**Affiliations:** 1 School of Dentistry, IMU University, Kuala Lumpur, MYS; 2 School of Dentistry, Adelaide University, Adelaide, AUS

**Keywords:** acrylic resin, color stability, digital photography, ocular prosthesis, outdoor weathering

## Abstract

Background and objective

Discoloration of ocular prostheses occurs unpredictably and can reduce their functional service life. Ensuring color stability is essential for maintaining long-term aesthetics and clinical performance. This study evaluated the color stability of acrylic ocular prostheses following short-term outdoor weathering using digital image analysis. A secondary aim was to quantify color changes within the weather-exposed group using digital photography with white balance calibration.

Materials and methods

Thirty custom acrylic ocular prostheses were fabricated and allocated to two groups: an outdoor-exposed (case) group and a group stored in darkness (control). The case group underwent outdoor weathering for three hours daily over one month. White balance color calibration was performed using a Macbeth color chart. L*, a*, and b* values were obtained from calibrated digital images in Adobe Photoshop (Adobe Inc., San Jose, CA, USA), and color changes (ΔE) were calculated. Independent-samples t-tests assessed differences between groups, while one-way ANOVA with Tukey post hoc analysis evaluated variation within the exposed group.

Results

After one month, ΔE values showed no significant difference between the case and control groups (p > 0.05). However, one-way ANOVA revealed significant color variation among the weather-exposed prostheses (p < 0.05).

Conclusions

Short-term exposure to tropical outdoor conditions was not associated with statistically significant differences in color stability compared with dark-room storage. The observed intragroup variability suggests that factors beyond environmental weathering contribute to color change. Appropriate fabrication techniques appear sufficient to maintain short-term color stability under tropical conditions.

## Introduction

The eyes are widely regarded as one of the most visually prominent facial features, and the absence of an eye often results in profound functional, emotional, and aesthetic consequences for affected individuals [1]. The loss of an eye may arise from congenital malformations, irreparable trauma, neoplasms, a painful blind eye, sympathetic ophthalmia, or the need for histopathological confirmation of a suspected diagnosis [2]. These conditions can impair patients’ ability to form social connections and diminish quality of life due to the combined psychosocial and aesthetic burden [3]. Prosthetic rehabilitation with an ocular prosthesis remains the gold standard for restoring facial appearance. Ocular prostheses may be prefabricated or custom-made, with the latter designed to replicate the color and form of the contralateral natural eye. However, color degradation, commonly occurring within one to five years, can compromise aesthetics and necessitate fabrication of a replacement prosthesis [4]. A variety of materials have been used in the fabrication of ocular prostheses, including polymethyl methacrylate, polyurethane elastomers, silicone elastomers, and urethane-backed medical-grade silicone [5,6]. Acrylic resin has favorable characteristics, including simplicity of use, comfort, versatility, and cost-effectiveness [5,6]. Acrylic resins can be precisely shaped to conform to the anatomical irregularities of the anophthalmic socket [6].

The longevity and aesthetic success of an ocular prosthesis depend largely on the color stability of the paints used during fabrication. Paints are susceptible to photodegradation when exposed to ultraviolet light or environmental stressors [7]. Previous studies have demonstrated that the degree of color change varies according to the type of paint used, with oil-based paints showing superior color stability under artificial ultraviolet light aging [7-9]. Advances in digital photography have enabled accurate image capture and objective color quantification through analysis of CIELAB coordinates (L*, a*, b*) [10]. Color differences (ΔE), calculated using the CIE 1976 formula [11], provide a standardized assessment of perceptible color change. Software-based post-processing white balance calibration using a Macbeth color chart enhances consistency across varying lighting environments [12]. Digital photography is therefore a practical and reliable method for evaluating color changes in ocular prostheses.

Previous research has largely focused on silicone-based prosthetic materials, with limited evidence concerning acrylic ocular prostheses subjected to natural outdoor weathering in tropical climates [13]. This study addresses this gap by evaluating the color stability of acrylic ocular prostheses exposed to Malaysian outdoor weather conditions.

The primary aim of this study was to compare color stability between weather-exposed acrylic ocular prostheses (case group) and prostheses stored in a dark environment (control group) using standardized photographic and color calibration protocols. The primary null hypothesis was that short-term tropical weathering would not affect color stability between groups. A secondary aim was to explore variation in color change among weather-exposed prostheses. The secondary null hypothesis was that no differences in color change would be observed among exposed specimens.

## Materials and methods

Study design

This was an in vitro experimental study comparing the color stability of acrylic ocular prostheses subjected to outdoor tropical weathering (case group) with prostheses stored in a dark environment (control group). The study employed a parallel-group design with equal allocation of specimens to each group.

Study setting and period

The study was conducted at the School of Dentistry, IMU, Kuala Lumpur, Malaysia. Prosthesis fabrication, photography, image analysis, and data collection were performed within the university facilities. The outdoor weathering experiment was conducted over a one-month period in December 2025.

Researcher training and standardization

Two researchers underwent supervised training with the project supervisor and an experienced dental technician to standardize all fabrication procedures. Training included mold fabrication, acrylic packing, polymerization, trimming, polishing, and scleral and iris color application. All prostheses were fabricated by the two trained researchers under direct supervision to ensure consistency.

Sample size calculation

Because no preliminary data were available regarding the expected difference in color change (ΔE) between weather-exposed and control prostheses, a formal power calculation based on a continuous outcome measure could not be performed. Consequently, this study was undertaken as an exploratory pilot investigation involving 30 prostheses, with 15 specimens allocated to each study group. The findings are intended to provide preliminary data for future sample size calculations and hypothesis-driven investigations.

Fabrication of acrylic ocular prostheses

A total of 30 acrylic ocular prostheses were fabricated. Heat-cured scleral polymer (J-510 Scleral Polymer, Technovent Ltd, Bridgend, UK) was combined with A2 and A3 acrylic resin shades (GC Unifast III, GC Corporation, Tokyo) to produce a white scleral base. The scleral polymer powder was mixed with monomer at a ratio of 1 scoop to 7 mL of monomer, supplemented with 2 mL each of A2 and A3 shades. The resin mixture was packed in a flask under hydraulic pressure and polymerized in water at 100 °C for three hours, following the manufacturer’s instructions. This procedure was repeated in three cycles to yield 30 ocular prostheses. Finishing and trimming were completed using fine acrylic burs.

Iris and scleral coloration

Oil-based pigments were used for intrinsic coloration. All painting was performed under identical natural indirect lighting. A mixture of red and blue pigments was blended with monopoly syrup added to monomer to adjust viscosity. Three layers of blue pigment were applied to the sclera and air-dried. Brown pigment was applied to form the iris region. Mixing ratios were recorded and replicated across all samples to ensure standardization.

Sample allocation and weathering protocol

**Figure 1:**
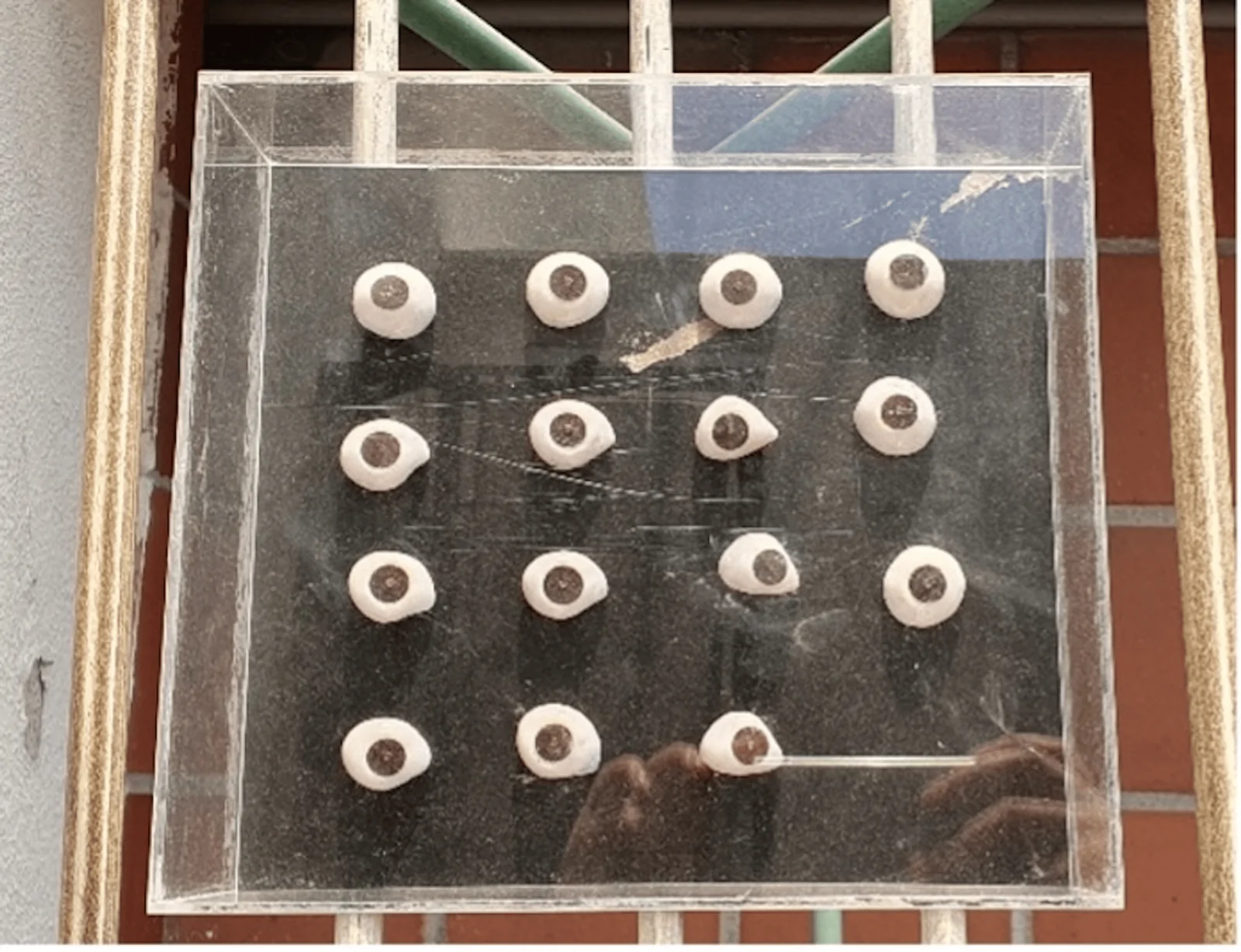
Prosthesis testing arrangement.

All 30 prostheses were sequentially numbered and allocated to two groups: (1) case group (n = 15): exposed to outdoor weathering and (2) control group (n = 15): stored in a dark room. Weathering exposure was conducted until 30 exposure days had been completed. Days with rainfall were excluded from the exposure schedule. Prostheses were arranged in five evenly spaced rows and stabilized with modeling wax within an uncovered clear acrylic box with a black base to prevent water stagnation (Figure 1). Control samples were stored in a dark room under an identical arrangement.

Photography and image acquisition

All samples were photographed before and after exposure. A digital camera (Canon EOS R, Canon Inc., Tokyo, Japan) with a macro lens (Canon EF 100 mm f/2.8L Macro IS USM) and ring flash (MR-14EX II) was used. A Macbeth color chart (SpyderCheckr 24; Datacolor, Trenton, NJ, USA) provided calibration. Camera settings were standardized: ISO 100, f/20, shutter speed 1/100, and color temperature 5900 K, with samples positioned 63 cm from the lens at a 90° angle. Room lighting and environmental conditions were identical for both photo sessions.

White balance calibration and color measurement

Images were transferred in RAW (CR3) format for analysis in Adobe Lightroom Classic (version 13.1, Adobe Inc., San Jose, CA, USA). A standardized workflow using SpyderCheckr 1.6 software was applied to calibrate white balance. Lightroom’s “Develop” module was used to obtain L*, a*, and b* values from eight constant measurement points on each prosthesis. The eight measurement locations (P1-P8) represented predefined regions distributed across the prosthesis surface and were applied consistently across all specimens and at both baseline and follow-up assessments.

Color difference calculation (ΔE)

Color change between pre- and post-exposure images was quantified using the CIE 1976 ΔE formula:

$\Delta E = \sqrt{(\Delta L)^2 + (\Delta a)^2 + (\Delta b)^2},$

where ΔL = difference in lightness, Δa = difference in the red-green axis, and Δb = difference in the yellow-blue axis. While CIEDE2000 may provide improved correlation with human visual perception, use of the CIE 1976 ΔE formula enabled direct comparison with previous ocular prosthesis investigations that employed the same metric.

Statistical analysis

Independent-samples t-tests were used to compare ΔE values between case and control groups. For the within-group analysis, mean ΔE values were calculated for each prosthesis from the eight predefined measurement locations. Because the eight measurement locations represented repeated observations within the same prosthesis and were not statistically independent, these measurements were averaged to generate a single prosthesis-level mean ΔE value. These prosthesis-level mean values were subsequently used for exploratory analysis of variation among weather-exposed specimens.

Within-group (case group) comparisons were assessed using a one-way ANOVA after confirming normality using the Shapiro-Wilk test. Post hoc comparisons were performed using Tukey’s honestly significant difference (HSD) test. Statistical significance was set at p ≤ 0.05. Analyses were conducted using IBM SPSS Statistics for Windows, version 28.0 (released 2021; IBM Corp., Armonk, NY, USA). The primary outcome measure was color change (ΔE) between baseline and one-month measurements. Eight predefined measurement locations were evaluated on each prosthesis.

## Results

**Figure 2:**
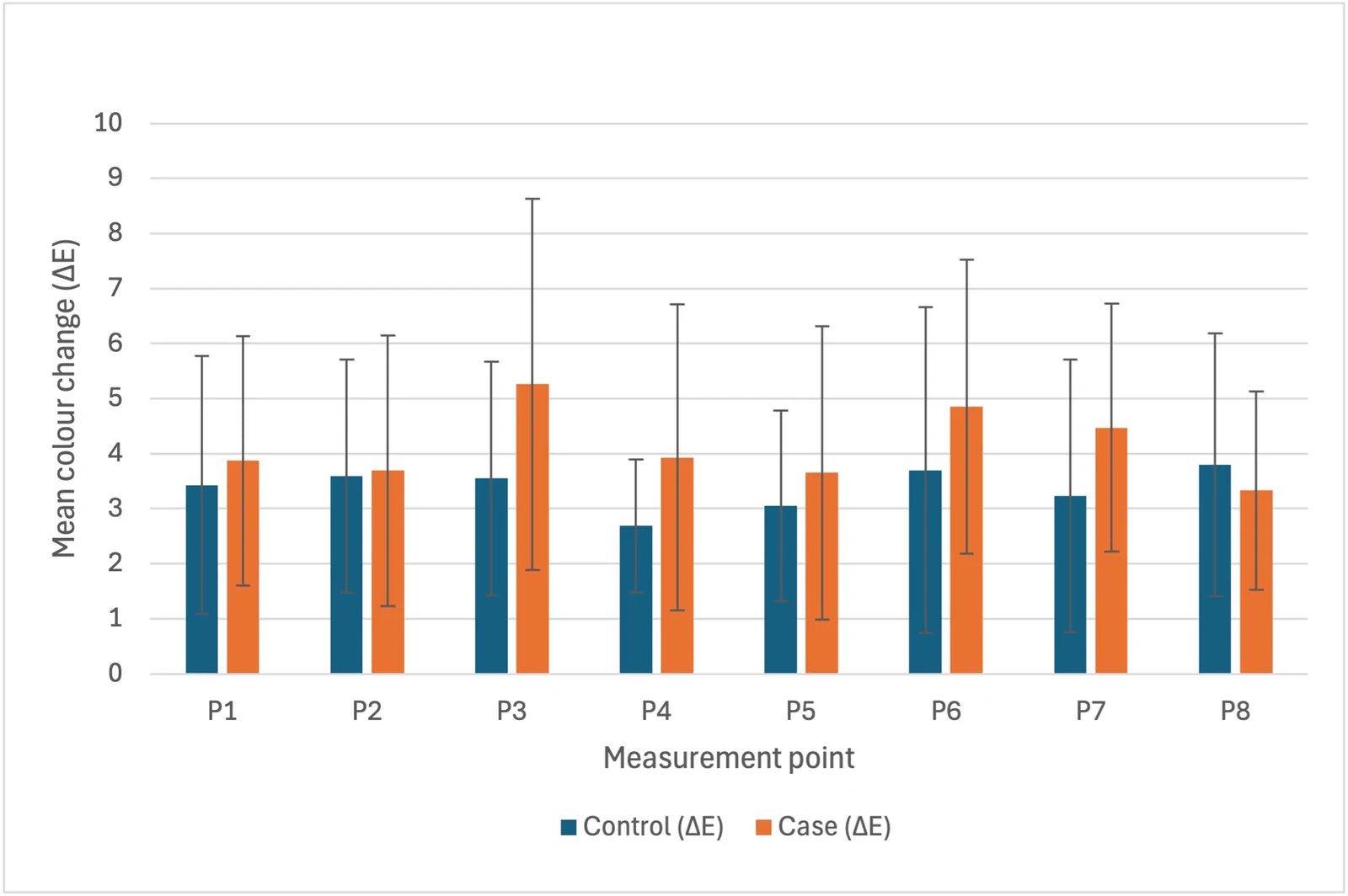
Mean color change (ΔE) ± SD for the eight measurement points in the control and weather-exposed groups.

Thirty ocular prostheses were evaluated, with 15 assigned to the weather-exposed group and 15 assigned to the control group. Mean color change (ΔE) values for each measurement point are presented in Table 1 and graphically illustrated in Figure 2. Across all measurement points, mean ΔE values ranged from 2.69 to 3.80 in the control group and from 3.33 to 5.26 in the weather-exposed group.

**Table 1:** Mean color change (ΔE) ± SD for the eight measurement points in the control and weather-exposed groups. p < 0.05 indicates statistical significance. Between-group comparisons were performed using independent-samples t-tests.

Point	Control (ΔE)	Case (ΔE)	t-value	p-value
P1	3.43 ± 2.34	3.87 ± 2.27	-0.52	0.605
P2	3.59 ± 2.12	3.69 ± 2.46	-0.12	0.906
P3	3.55 ± 2.12	5.26 ± 3.37	-1.66	0.11
P4	2.69 ± 1.21	3.93 ± 2.78	-1.59	0.13
P5	3.05 ± 1.73	3.65 ± 2.66	-0.73	0.471
P6	3.70 ± 2.96	4.85 ± 2.67	-1.12	0.274
P7	3.23 ± 2.48	4.47 ± 2.25	-1.44	0.163
P8	3.80 ± 2.39	3.33 ± 1.80	0.61	0.548

No statistically significant differences were identified between weather-exposed and control prostheses at any measurement location (all p > 0.05). The largest difference was observed at P3, where the weather-exposed group demonstrated a higher mean ΔE value than the control group; however, this difference was not statistically significant (p = 0.110).

**Figure 3:**
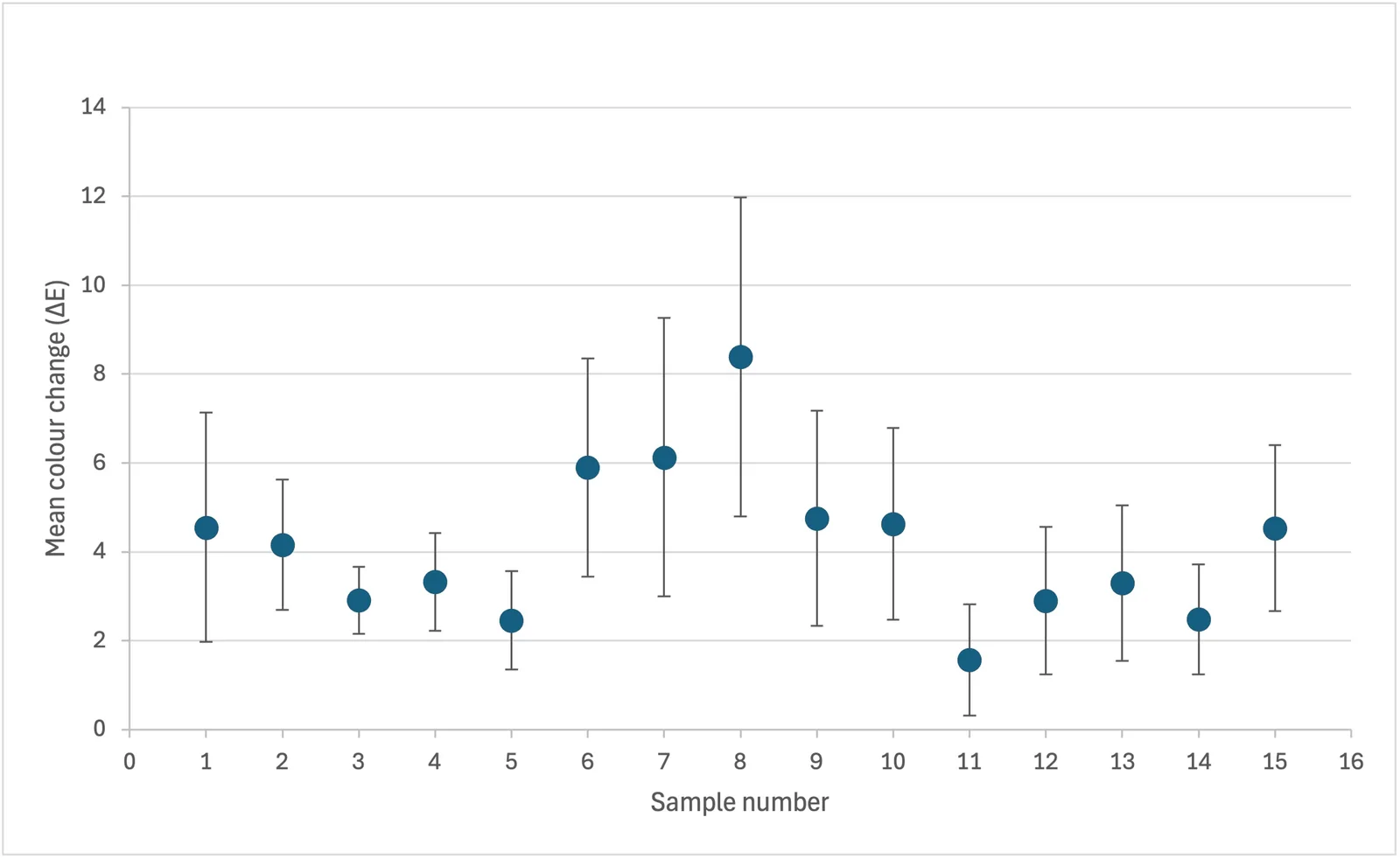
Mean color change (ΔE) ± SD across the 15 weather-exposed samples. One-way ANOVA demonstrated significant variation among samples (F = 5.761, p < 0.001).

Color variation among the 15 weather-exposed prostheses is summarized in Table 2 and Figure 3. Mean ΔE values ranged from 1.57 ± 1.25 to 8.39 ± 3.59. Significant variation in mean ΔE values was observed among the weather-exposed prostheses (one-way ANOVA: F = 5.761, p < 0.001).

**Table 2:** Mean color change (ΔE) ± SD and 95% CIs for the 15 weather-exposed samples. * p < 0.05 indicates statistical significance. Data are presented as mean ± SD and 95% CIs. Statistical analysis was performed using one-way ANOVA. Differences among samples were assessed using one-way ANOVA (F = 5.761, p < 0.001).

Sample	Mean ΔE ± SD	95% CI	p-value
1	4.55 ± 2.58	2.39-6.70	<0.001*
2	4.16 ± 1.47	2.93-5.39	
3	2.91 ± 0.75	2.29-3.53	
4	3.33 ± 1.10	2.41-4.24	
5	2.46 ± 1.11	1.54-3.39	
6	5.90 ± 2.46	3.84-7.95	
7	6.13 ± 3.13	3.51-8.75	
8	8.39 ± 3.59	5.39-11.39	
9	4.76 ± 2.42	2.74-6.79	
10	4.63 ± 2.16	2.83-6.44	
11	1.57 ± 1.25	0.53-2.61	
12	2.90 ± 1.66	1.51-4.30	
13	3.30 ± 1.75	1.84-4.77	
14	2.48 ± 1.24	1.44-3.51	
15	4.53 ± 1.87	2.97-6.09	

Post hoc Tukey’s HSD analysis identified several significant pairwise differences, primarily involving Sample 8, which exhibited the greatest mean color change (Table 3).

**Table 3:** Post hoc pairwise comparisons among samples using Tukey’s HSD test. * Indicates p < 0.05 based on Tukey’s HSD test. Dependent variable: ∆E HSD, honestly significant difference

Samples (I)	Samples (J)	Mean difference (I-J)	SE	Sig.	95% CI	
					Lower bound	Upper bound
1	2	0.39	1.03	1.00	-3.18	3.96
	3	1.64	1.03	0.956	-1.93	5.21
	4	1.22	1.03	0.997	-2.35	4.8
	5	2.09	1.03	0.776	-1.48	5.66
	6	-1.35	1.03	0.992	-4.92	2.23
	7	-1.58	1.03	0.968	-5.15	1.99
	8	-3.84*	1.03	0.023*	-7.41	-0.27
	9	-0.21	1.03	1.00	-3.79	3.36
	10	-0.08	1.03	1.00	-3.65	3.49
	11	2.98	1.03	0.214	-0.59	6.55
	12	1.64	1.03	0.955	-1.93	5.22
	13	1.25	1.03	0.996	-2.32	4.82
	14	2.07	1.03	0.785	-1.5	5.65
	15	0.02	1.03	1.00	-3.55	3.59
2	1	-0.39	1.03	1.00	-3.96	3.18
	3	1.25	1.03	0.996	-2.32	4.82
	4	0.83	1.03	1.00	-2.74	4.41
	5	1.7	1.03	0.943	-1.88	5.27
	6	-1.74	1.03	0.932	-5.31	1.83
	7	-1.97	1.03	0.839	-5.55	1.6
	8	-4.23*	1.03	0.006*	-7.8	-0.66
	9	-0.6	1.03	1.00	-4.18	2.97
	10	-0.47	1.03	1.00	-4.05	3.1
	11	2.59	1.03	0.436	-0.98	6.16
	12	1.25	1.03	0.996	-2.32	4.83
	13	0.86	1.03	1.00	-2.72	4.43
	14	1.68	1.03	0.947	-1.89	5.25
	15	-0.37	1.03	1.00	-3.94	3.2
3	1	-1.64	1.03	0.956	-5.21	1.93
	2	-1.25	1.03	0.996	-4.82	2.32
	4	-0.42	1.03	1.00	-3.99	3.16
	5	0.45	1.03	1.00	-3.12	4.02
	6	-2.99	1.03	0.212	-6.56	0.59
	7	-3.22	1.03	0.125	-6.79	0.35
	8	-5.48*	1.03	<0.001*	-9.05	-1.91
	9	-1.85	1.03	0.892	-5.42	1.72
	10	-1.72	1.03	0.936	-5.29	1.85
	11	1.34	1.03	0.993	-2.23	4.92
	12	0	1.03	1.00	-3.57	3.58
	13	-0.39	1.03	1.00	-3.96	3.18
	14	0.43	1.03	1.00	-3.14	4.01
	15	-1.62	1.03	0.961	-5.19	1.95
4	1	-1.22	1.03	0.997	-4.8	2.35
	2	-0.83	1.03	1.00	-4.41	2.74
	3	0.42	1.03	1.00	-3.16	3.99
	5	0.86	1.03	1.00	-2.71	4.44
	6	-2.57	1.03	0.45	-6.14	1.00
	7	-2.81	1.03	0.303	-6.38	0.77
	8	-5.06*	1.03	<0.001*	-8.64	-1.49
	9	-1.44	1.03	0.986	-5.01	2.14
	10	-1.31	1.03	0.994	-4.88	2.27
	11	1.76	1.03	0.926	-1.81	5.33
	12	0.42	1.03	1.00	-3.15	3.99
	13	0.02	1.03	1.00	-3.55	3.6
	14	0.85	1.03	1.00	-2.72	4.42
	15	-1.2	1.03	0.998	-4.77	2.37
5	1	-2.09	1.03	0.776	-5.66	1.48
	2	-1.7	1.03	0.943	-5.27	1.88
	3	-0.45	1.03	1.00	-4.02	3.12
	4	-0.86	1.03	1.00	-4.44	2.71
	6	-3.43	1.03	0.073	-7.01	0.14
	7	-3.67*	1.03	0.038*	-7.24	-0.1
	8	-5.93*	1.03	<0.001*	-9.5	-2.36
	9	-2.3	1.03	0.638	-5.87	1.27
	10	-2.17	1.03	0.725	-5.74	1.4
	11	0.89	1.03	1.00	-2.68	4.47
	12	-0.44	1.03	1.00	-4.02	3.13
	13	-0.84	1.03	1.00	-4.41	2.73
	14	0	1.03	1.00	-3.59	3.56
	15	-2.07	1.03	0.789	-5.64	1.51
6	1	1.35	1.03	0.992	-2.23	4.92
	2	1.74	1.03	0.932	-1.83	5.31
	3	2.99	1.03	0.212	-0.59	6.56
	4	2.57	1.03	0.45	-1	6.14
	5	3.43	1.03	0.073	-0.14	7.01
	7	-0.23	1.03	1.00	-3.81	3.34
	8	-2.49	1.03	0.503	-6.07	1.08
	9	1.13	1.03	0.999	-2.44	4.71
	10	1.26	1.03	0.996	-2.31	4.84
	11	4.33*	1.03	0.005*	0.76	7.9
	12	2.99	1.03	0.210	-0.58	6.56
	13	2.59	1.03	0.434	-0.98	6.17
	14	3.42	1.03	0.076	-0.15	6.99
	15	1.37	1.03	0.991	-2.2	4.94
7	1	1.58	1.03	0.968	-1.99	5.15
	2	1.97	1.03	0.839	-1.6	5.55
	3	3.22	1.03	0.125	-0.35	6.79
	4	2.81	1.03	0.303	-0.77	6.38
	5	3.67*	1.03	0.038*	0.1	7.24
	6	0.23	1.03	1.00	-3.34	3.81
	8	-2.26	1.03	0.667	-5.83	1.31
	9	1.37	1.03	0.991	-2.2	4.94
	10	1.5	1.03	0.979	-2.07	5.07
	11	4.56*	1.03	0.002*	0.99	8.14
	12	3.23	1.03	0.124	-0.35	6.8
	13	2.83	1.03	0.29	-0.74	6.4
	14	3.65*	1.03	0.040*	0.08	7.23
	15	1.6	1.03	0.964	-1.97	5.18
8	1	3.84*	1.03	0.023*	0.27	7.41
	2	4.23*	1.03	0.006*	0.66	7.8
	3	5.48*	1.03	<0.001*	1.91	9.05
	4	5.06*	1.03	<0.001*	1.49	8.64
	5	5.93*	1.03	<0.001*	2.36	9.5
	6	2.49	1.03	0.503	-1.08	6.07
	7	2.26	1.03	0.667	-1.31	5.83
	9	3.63*	1.03	0.043*	0.05	7.2
	10	3.76*	1.03	0.029*	0.19	7.33
	11	6.82*	1.03	<0.001*	3.25	10.39
	12	5.48*	1.03	<0.001*	1.91	9.06
	13	5.09*	1.03	<0.001*	1.52	8.66
	14	5.91*	1.03	<0.001*	2.34	9.49
	15	3.86*	1.03	0.021*	0.29	7.43
9	1	0.21	1.03	1.00	-3.36	3.79
	2	0.6	1.03	1.00	-2.97	4.18
	3	1.85	1.03	0.892	-1.72	5.42
	4	1.44	1.03	0.986	-2.14	5.01
	5	2.3	1.03	0.638	-1.27	5.87
	6	-1.13	1.03	0.999	-4.71	2.44
	7	-1.37	1.03	0.991	-4.94	2.2
	8	-3.63*	1.03	0.043*	-7.2	-0.05
	10	0.13	1.03	1.00	-3.44	3.7
	11	3.19	1.03	0.133	-0.38	6.77
	12	1.86	1.03	0.891	-1.72	5.43
	13	1.46	1.03	0.984	-2.11	5.03
	14	2.29	1.03	0.648	-1.29	5.86
	15	0.23	1.03	1.00	-3.34	3.81
10	1	0.08	1.03	1.00	-3.49	3.65
	2	0.47	1.03	1.00	-3.1	4.05
	3	1.72	1.03	0.936	-1.85	5.29
	4	1.31	1.03	0.994	-2.27	4.88
	5	2.17	1.03	0.725	-1.4	5.74
	6	-1.26	1.03	0.996	-4.84	2.31
	7	-1.5	1.03	0.979	-5.07	2.07
	8	-3.76*	1.03	0.029*	-7.33	-0.19
	9	-0.13	1.03	1.00	-3.7	3.44
	11	3.06	1.03	0.179	-0.51	6.64
	12	1.73	1.03	0.935	-1.85	5.3
	13	1.33	1.03	0.993	-2.24	4.9
	14	2.15	1.03	0.735	-1.42	5.73
	15	0.1	1.03	1.00	-3.47	3.68
11	1	-2.98	1.03	0.214	-6.55	0.59
	2	-2.59	1.03	0.436	-6.16	0.98
	3	-1.34	1.03	0.993	-4.92	2.23
	4	-1.76	1.03	0.926	-5.33	1.81
	5	-0.89	1.03	1.00	-4.47	2.68
	6	-4.33*	1.03	0.005*	-7.9	-0.76
	7	-4.56*	1.03	0.002*	-8.14	-0.99
	8	-6.82*	1.03	<0.001*	-10.39	-3.25
	9	-3.19	1.03	0.133	-6.77	0.38
	10	-3.06	1.03	0.179	-6.64	0.51
	12	-1.34	1.03	0.993	-4.91	2.23
	13	-1.73	1.03	0.933	-5.31	1.84
	14	-0.91	1.03	1.00	-4.48	2.66
	15	-2.96	1.03	0.224	-6.53	0.61
12	1	-1.64	1.03	0.955	-5.22	1.93
	2	-1.25	1.03	0.996	-4.83	2.32
	3	0	1.03	1.00	-3.58	3.57
	4	-0.42	1.03	1.00	-3.99	3.15
	5	0.44	1.03	1.00	-3.13	4.02
	6	-2.99	1.03	0.21	-6.56	0.58
	7	-3.23	1.03	0.124	-6.8	0.35
	8	-5.48*	1.03	<0.001*	-9.06	-1.91
	9	-1.86	1.03	0.891	-5.43	1.72
	10	-1.73	1.03	0.935	-5.3	1.85
	11	1.34	1.03	0.993	-2.23	4.91
	13	-0.4	1.03	1.00	-3.97	3.18
	14	0.43	1.03	1.00	-3.14	4
	15	-1.62	1.03	0.960	-5.19	1.95
13	1	-1.25	1.03	0.996	-4.82	2.32
	2	-0.86	1.03	1.00	-4.43	2.72
	3	0.39	1.03	1.00	-3.18	3.96
	4	-0.02	1.03	1.00	-3.6	3.55
	5	0.84	1.03	1.00	-2.73	4.41
	6	-2.59	1.03	0.434	-6.17	0.98
	7	-2.83	1.03	0.29	-6.4	0.74
	8	-5.09*	1.03	<0.001*	-8.66	-1.52
	9	-1.46	1.03	0.984	-5.03	2.11
	10	-1.33	1.03	0.993	-4.9	2.24
	11	1.73	1.03	0.933	-1.84	5.31
	12	0.4	1.03	1.00	-3.18	3.97
	14	0.83	1.03	1.00	-2.75	4.4
	15	-1.23	1.03	0.997	-4.8	2.35
14	1	-2.07	1.03	0.785	-5.65	1.5
	2	-1.68	1.03	0.947	-5.25	1.89
	3	-0.43	1.03	1.00	-4.01	3.14
	4	-0.85	1.03	1.00	-4.42	2.72
	5	0.02	1.03	1.00	-3.56	3.59
	6	-3.42	1.03	0.076	-6.99	0.15
	7	-3.65*	1.03	0.040*	-7.23	-0.08
	8	-5.91*	1.03	<0.001*	-9.49	-2.34
	9	-2.29	1.03	0.648	-5.86	1.29
	10	-2.15	1.03	0.735	-5.73	1.42
	11	0.91	1.03	1.00	-2.66	4.48
	12	-0.43	1.03	1.00	-4	3.14
	13	-0.83	1.03	1.00	-4.4	2.75
	15	-2.05	1.03	0.797	-5.62	1.52
15	1	-0.02	1.03	1.00	-3.59	3.55
	2	0.37	1.03	1.00	-3.2	3.94
	3	1.62	1.03	0.961	-1.95	5.19
	4	1.2	1.03	0.998	-2.37	4.77
	5	2.07	1.03	0.789	-1.51	5.64
	6	-1.37	1.03	0.991	-4.94	2.2
	7	-1.6	1.03	0.964	-5.18	1.97
	8	-3.86*	1.03	0.021*	-7.43	-0.29
	9	-0.23	1.03	1.00	-3.81	3.34
	10	-0.1	1.03	1.00	-3.68	3.47
	11	2.96	1.03	0.224	-0.61	6.53
	12	1.62	1.03	0.960	-1.95	5.19
	13	1.23	1.03	0.997	-2.35	4.8
	14	2.05	1.03	0.797	-1.52	5.62

## Discussion

No significant differences in color change (ΔE) were detected between the weather-exposed and dark-room control groups; therefore, the null hypothesis was not rejected. Nevertheless, both groups exhibited measurable color alteration over the one-month period, despite differing environmental conditions. This is consistent with prior evidence that pigment and polymer systems can undergo intrinsic and extrinsic degradation, with matrix alterations within the material itself (intrinsic) and stain absorption/adsorption from the environment (extrinsic) each contributing to chromatic drift [14]. Extrinsic stressors such as ultraviolet radiation, humidity, and heat may accelerate polymer deterioration and paint degradation by promoting chain scission and loss of properties, with rates increasing alongside temperature [14,15]. These mechanisms provide a plausible explanation for the observed color changes in both exposed and control samples, and they also help account for within-group variability among exposed specimens, where outdoor conditions are not the sole determinant of color change. The observed variability among weather-exposed prostheses should not be interpreted as evidence that environmental weathering was the direct cause of the differences observed. Factors including pigment application variability, prosthesis geometry, material characteristics, and measurement variability may also have contributed to the observed differences.

The exposure protocol in this study (three hours of daily outdoor exposure over one month) differs from earlier reports that used longer durations or continuous accelerated aging [16]. Our design was intended to approximate a realistic daily exposure window rather than continuous outdoor conditions, acknowledging that patients are unlikely to experience uninterrupted sun exposure. Compared with studies on silicone elastomers exposed in hot and humid climates, which commonly report ΔE values exceeding clinical acceptability thresholds [13,17], the acrylic prostheses in the present study did not show significant between-group differences after one month. Material selection and measurement methodology may partly explain these discrepancies. Acrylic may exhibit different weathering behavior than silicone systems, and our workflow used standardized digital photography with white balance calibration rather than spectrophotometry adopted elsewhere [13,18]. In addition, the CIE 1976 ΔE formula was retained to facilitate comparison with the majority of previously published ocular prosthesis studies that used the same metric.

The pigment system may also influence outcomes. Bunker et al. reported lower ΔE for blue irises relative to brown and green in acrylic ocular prostheses, aligning with the modest post-exposure differences observed here, where blue formed the basis of scleral coloration [18]. While this suggests comparatively greater stability of blue hues, other literature indicates that certain blue paints can be less stable than sepia/brown tones depending on resin interactions, residual monomer, and UV exposure [7,19,20]. The present study cannot resolve these conflicting findings; however, it underscores the need to examine paint shade and layer thickness as cofactors, especially given potential viscosity changes during painting (e.g., thickening as monopoly syrup and monomer evaporate), which may yield variable pigment film thickness and, consequently, variable ΔE among samples.

Methodological differences further contextualize our findings. Unlike accelerated protocols that tightly control irradiance, temperature, and humidity [9,14,15], our experiment relied on natural tropical conditions. Angle of solar incidence, micro-shading within the clear acrylic enclosure, and localized heating may have produced nonuniform exposure across curved prosthetic surfaces, contributing to the significant ANOVA result within the exposed group and the observed dispersion. Surface geometry likely played a role, as regions with greater concavity or inclination can receive uneven illumination during photography, potentially amplifying apparent color differences despite standardized camera-to-sample distance and flash settings.

Although the present findings were derived from an in vitro experimental model, they suggest that periodic monitoring of color stability may be useful when considering prosthesis replacement and aesthetic maintenance in clinical practice. Future studies should consider larger samples and longer monitoring periods, flatter or standardized test geometries to minimize lighting artifacts, controlled accelerated weathering to complement natural exposure, and systematic comparison of scleral shades and pigment types to clarify shade-dependent stability.

Finally, prior work comparing polymerization cycles has shown clinically unacceptable color changes (ΔE > 3.3) across various curing schedules for scleral resins, with notable fluctuations between short and long cycles [21]. Our three-hour cycle is closest to a short-cycle condition, and although the present study did not detect between-group differences after one month, the within-group dispersion we observed mirrors the fluctuation patterns reported in that literature. Together, these data suggest that color stability is a multifactorial outcome shaped by base material, pigment system, processing, geometry, and exposure history, elements that should be jointly optimized to maintain long-term aesthetics in ocular prostheses.

This study has several limitations. First, the observation period was limited to one month and may not reflect the long-term clinical behavior of ocular prostheses. Second, color assessment was performed using calibrated digital photography rather than spectrophotometry, which remains the reference standard for objective color measurement. Although a standardized photographic workflow was employed, potential sources of error remain, including white balance calibration variability, shadowing, and differences in illumination across curved prosthesis surfaces. Third, natural outdoor weathering conditions introduce unavoidable variability in temperature, humidity, and ultraviolet exposure. Environmental variables such as ultraviolet irradiance, ambient temperature, and humidity were not continuously monitored throughout the outdoor exposure period and therefore could not be incorporated into the analysis. Fourth, the custom prosthesis shapes and minor variation in pigment layer thickness may have contributed to within-group variability in ΔE measurements. Fifth, the sample size was modest, and the study should be considered exploratory. Finally, color measurements were obtained from predefined measurement points, which may not fully capture the overall color behavior of the prosthesis surface.

## Conclusions

Over one month, acrylic ocular prostheses showed no significant between-group differences in color change (ΔE) between weather-exposed and dark-room conditions, although both groups exhibited modest color changes, reflecting multifactorial influences on color stability. These findings suggest that acrylic ocular prostheses demonstrated color stability under the specific one-month outdoor exposure conditions evaluated in this in vitro study. Further investigations incorporating longer observation periods, controlled environmental conditions, and clinical assessments are required before broader conclusions regarding long-term clinical performance can be drawn.
